# The role of the endocannabinoid system in female reproductive tissues

**DOI:** 10.1186/s13048-018-0478-9

**Published:** 2019-01-15

**Authors:** O’ Llenecia S. Walker, Alison C. Holloway, Sandeep Raha

**Affiliations:** 1 Department of Pediatrics, and the Graduate Program in Medical Sciences, McMaster University, 1280 Main Street West, HSC 3N11H, Hamilton, ON L8S 4K1 Canada; 2 Department of Obstetrics and Gynecology and the Graduate Program in Medical Sciences, McMaster University, 1280 Main Street West, HSC 3N52A, Hamilton, ON L8S 4K1 Canada

**Keywords:** Female reproduction, Endocannabinoid system, Ovary, Cannabis, PCOS, Ovarian cancer

## Abstract

There has been increasing interest in the role of endocannabinoids as critical modulators of the female reproductive processes. Endocannabinoids are natural ligands of cannabinoid, vanilloid, and peroxisome proliferator-activated receptors. Together with their receptors, enzymes and downstream signaling targets, they form the endocannabinoid system (ECS). While the ECS is known to modulate pain and neurodevelopment, it is also known to impact the female reproductive system where it affects folliculogenesis, oocyte maturation, and ovarian endocrine secretion. In addition, the ECS affects oviductal embryo transport, implantation, uterine decidualization and placentation. There is a complex interplay between the ECS and the hypothalamic-pituitary-ovarian axis, and an intricate crosstalk between the ECS and steroid hormone production and secretion. Exogenous cannabinoids, derived from plants such as *Cannabis sativa*, are also ligands for cannabinoid receptors. These have been shown to have clinical outcomes related to ECS dysregulation, including multiple sclerosis, Alzheimer’s disease, and amyotrophic lateral sclerosis, along with adverse effects on female reproduction. The aim of this review is to describe and discuss data from human, animal, and in vitro studies that support the important role of the endocannabinoid system in female reproductive tissues and processes. In particular, we will discuss some of the mechanisms by which endocannabinoid signaling can affect ovarian function in both physiological and pathophysiological states.

## Overview of the endocannabinoid system

Cannabinoids are a class of compounds that are plant-derived (phytocannabinoids, i.e. from *Cannabis sativa*), made naturally within the body, and chemically manufactured. For the purpose of this review, where the term “cannabinoids” is used, it will refer to the general class of compounds, unless specified otherwise. Endocannabinoids (endogenous cannabinoids) are unsaturated fatty acid derivatives with wide distribution in the human body [1–3]. The two most extensively studied endocannabinoids are N-arachidonoylethanolamine (anandamide; AEA) and 2-arachidonoylglycerol (2-AG). AEA was the first endocannabinoid to be discovered [4] and is an important intermediate in lipid metabolism [5]. Its name was derived from the Sanskrit word “*ananda*” which means inner bliss, describing the euphoric effects of this ligand [5, 6]. Physiologically, it is produced ubiquitously [5] with the greatest tissue concentrations found in the brain [6]. Under most physiological conditions, 2-AG concentrations are much higher than that of AEA [5]. Interestingly, these endocannabinoids are not stored within cells, but are thought to be manufactured “on demand” from membrane phospholipid precursors [7, 8]. However, recent reports challenge this view suggesting that they may be stored intracellularly within lipid droplets referred to as adiposomes, thus allowing for intracellular accumulation [5, 9]. Others suggest that catabolic enzymes at the surface of adiposomes quickly lead to endocannabinoid degradation [7]. In light of this, sequestering of endocannabinoids in adiposomes may prolong their half-life (hours rather than minutes), allowing them time to trigger nuclear receptors [7].

AEA and 2-AG are known to bind to and activate two G-protein coupled receptors (GPCR): cannabinoid receptor 1 (CB1) and CB2. Both receptor isoforms are ubiquitously expressed, with CB1 found more predominantly in the central nervous system and CB2 found largely in cells of the immune system [5, 10, 11]. The receptor actions of AEA and 2-AG are mimicked by the exogenous cannabinoid Δ-9-tetrahydrocannabinol (THC), the primary psychoactive component of cannabis [4, 12]. In fact, it was the discovery in the 1980s that THC could bind to receptors in the brain that led researchers to discover AEA, the prototypical endocannabinoid [13].

### AEA: Synthesis, transport, and degradation

AEA acts as a partial agonist at CB1 and as a weak/partial agonist at CB2 [5]. Interestingly, AEA is reported to demonstrate promiscuous binding activity [11], as it can trigger various signaling pathways via a number of different receptors, both extracellularly at CB1and CB2, intracellularly at the transient receptor potential vanilloid-1 (TRPV1) channel, and in the nucleus via the peroxisome proliferator-activated receptors (PPARs) [7]. More recently, studies have suggested that AEA may also act at the level of the mitochondria via CB1 receptors situated on the mitochondrial outer membrane [14].

The biosynthesis of AEA occurs in two steps. First, AEA is released from phospholipid precursors in the plasma membrane to a phosphatidylethanolamine, leading to the formation of N-acylphosphatidylethanolamine (NAPE). In the second step, a type D phospholipase (NAPE-PLD) catalyzes the formation of AEA from its NAPE precursor [5]. AEA is then rapidly taken up by cells [8, 15]. Movement of AEA across the phospholipid bilayer is thought to occur by simple diffusion or endocytosis [8, 15] since AEA is uncharged and lipid soluble. Other studies strongly suggest the involvement of a putative endocannabinoid membrane transporter (EMT) [8, 16, 17] which allows AEA to be rapidly shuttled to its intracellular targets [8, 15]. Some of the intracellular targets for AEA identified to date include AEA intracellular binding proteins (AIBPs), namely albumin, heat shock protein 70 (Hsp70) and fatty acid binding protein-5 and -7 (FABP-5 and -7) [8]. It has been proposed that FABPs are principally involved in AEA trafficking and breakdown, as the use of a novel reversible FABP inhibitor, BMS309403, partially reduced AEA uptake [15]. Ultimately, AEA is broken down by intracellular hydrolases into ethanolamine and arachidonic acid [5, 7]. A key regulator of AEA activity is the serine hydrolase fatty acid amide hydrolase (FAAH) which is bound to intracellular membranes, particularly the endoplasmic reticulum (ER) and nuclear membrane [7].

### 2-AG: Synthesis, transport, and degradation

Belonging to the monoacylglycerol (MAG) family of endocannabinoids, 2-AG acts equally at CB1 and CB2 as a full potent agonist, but it has not been shown to act at the TRPV1 receptor [5, 11]. Tissue levels of 2-AG are markedly higher than that of AEA within the same tissue [5]. Coupled with its full agonistic activity at cannabinoid receptors, it has been proposed as the primary endogenous agonist of both CB1 and CB2 [8].

The biosynthesis of 2-AG involves the combined action of two membrane-bound enzymes: phospholipase C (PLC) and diacylglycerol lipase (DAGL) [5]. Unlike AEA, only a few studies have investigated the mechanisms underlying the rapid cellular uptake of 2-AG, which is surprising given that it is more abundant than AEA [8]. While the mechanism(s) may be dependent upon the cell type [8, 16, 18], the most likely routes of entry for 2-AG may be via endocytosis, simple diffusion, the same EMT as AEA or other transporters [8, 16].

Once within the cytosol, 2-AG becomes a substrate for the chief catalytic enzyme monoacylglycerol lipase (MAGL), associated with the inner membrane, which degrades 2-AG to glycerol and arachidonic acid [8]. In addition to MAGL, two other 2-AG hydrolases have been identified: α,β-hydrolase-6 and -12 (ABHD-6 and -12), which are integral cell membrane proteins and thought to share the catalytic triad with MAGL [8]. In addition to the consensus regarding the putative degradative enzymes, these prototypical endocannabinoids may also be degraded via oxidation by cyclooxygenase (COX), lipoxygenase (LOX), or cytochrome P450 [5]. These and other biosynthetic and degradative pathways for AEA and 2-AG are discussed at length in Fezza et al [5].

### THC and other ligands

THC was the first exogenous ligand of cannabinoid receptors to be discovered [13]. The physiological effects of THC are clinically concerning and need to be well delineated. Since this compound is highly lipophilic [5, 12, 19], it can be readily sequestered into adipose tissue, resulting in a rapid decrease in plasma concentrations [19], followed by a slow release into circulation over extended periods of time [12]. This tissue distribution permits longer-lasting stimulation of the cannabinoid receptors. This is unlike that of the locally released endocannabinoids AEA and 2-AG, which are rapidly inactivated by their transporters and hydrolases [12].

Endogenously, other fatty acid derived compounds, including N-arachidonoyldopamine (NADA), 2-arachidonoylglycerylether (noladin ether) and O-arachidonoyl ethanolamine (virodhamine) have also been identified as endocannabinoids, although information on their function and biological relevance is somewhat limited [4, 5, 10]. Interestingly, a novel group of ligands has been identified, referred to as retro-anandamides, which are characterized by a reversal in the positions of the carbonyl and the amido groups [20]. While these compounds demonstrate reduced affinity for CB1 and CB2 receptors as compared to AEA, they are resistant to FAAH catabolism resulting in increased stability relative to AEA [20]. These same authors, in an earlier report, identified the first metabolically stable AEA analogue, (R)-methanandamide, which exhibited significantly greater affinity for CB1 and resistance to FAAH degradation when compared to AEA [20]. Despite their presence, these and other ligands have received less scientific attention than AEA and 2-AG, perhaps due to the difficulty involved in isolating them from biological tissues [5].

### Cannabinoid receptors

CB1 and CB2 belong to a large superfamily of seven-transmembrane spanning GPCRs [4]. AEA or 2-AG ligand binding to either CB1 or CB2 leads to multiple signal transduction mechanisms, including the inhibition of adenylate cyclase [4], a common target for activated G proteins, and consequent decrease in intracellular cAMP levels, increased potassium influx, and/or inhibition of certain calcium channels, thus reducing calcium influx [21, 22]. The intracellular signals arising from these cascades subsequently leads to the regulation of growth, proliferation, and/or differentiation [6].

### The CB1 receptor

CB1 receptors are found mostly within the central nervous system [22]. Peripherally, CB1 receptors have been identified in the spleen, heart, adrenal gland, ovaries, endometrium, testes, among others [1, 22]. Furthermore CB1 receptors have also been localized intracellularly on the mitochondrial outer membrane [14]. Mitochondria regulate the energy demands of the cell, thus compromises in its function, from aberrant cannabinoid signaling [23, 24], will deregulate energy metabolism [25]. For example, THC induced mitochondrial dysfunction has been associated with pathologies such as stroke [26]. Mechanistically, mitochondrial CB1 receptors are thought to modulate complex I activity via a process involving soluble adenylyl cyclase [27]. Since mitochondrial function controls apoptosis, disruption of this role can impact the process of producing quality gametes, subsequently interfering with embryogenesis and lead to the production poor quality embryonic stem cells [28]. Furthermore, ovarian ageing is also associated with increased accumulation of mitochondrial DNA mutations which are likely to affect mitochondrial biogenesis and impact oocyte quality [29]. Additionally, placental oxidative stress is also linked to mitochondrial dysfunction [30] and may impact vascular remodeling in the placenta [31] which is important for tissue oxygenation and organ function. Dysregulation of placental vascular development can result in a number of adverse pregnancy outcomes such as intrauterine growth restriction and preeclampsia [32, 33]. CB1 receptors are also found in the hypothalamus, the central regulator of energy homeostasis, further implicating a role for the ECS in energy balance. Moreover, the preoptic area of the hypothalamus contain CB1 receptors, from which secretory neurons for gonadotropin-releasing hormone (GnRH) are located [34].

### The CB2 receptor

Like the CB1 receptor, CB2 is also a G protein coupled receptor demonstrating 44% sequence homology to CB1 [6, 22]. CB2 receptors are predominantly found peripherally within cells of the immune system, such as lymphocytes and macrophages [23]. El-Talatini et al confirmed the presence of CB2 receptors in the ovarian cortex, ovarian medulla, and ovarian follicles from human samples [1], which followed a similar staining pattern as CB1 in the same tissues [1]. However, Wang et al found that in murine oocytes, the action of endocannabinoids were mediated by CB1 receptor activation, not that of CB2. [35]. This interspecies difference highlights the importance of utilizing human reproductive tissues as a means to study the impact of the ECS on its function/dysregulation [1].

### Other receptors

Endocannabinoids are thought to also target a number of other orphan receptors. These include the GPR55 (also known as CB3) and GPR119 receptors which have signaling mechanisms distinct from CB1/CB2 [3, 16, 36]. Other receptors targeted by endocannabinoids include TRPV1, cytosolic target for AEA; and nuclear PPAR. Pertwee et al provide a detailed review of these other receptors and their pharmacology [10].

## ECS and reproduction

**Fig. 1:**
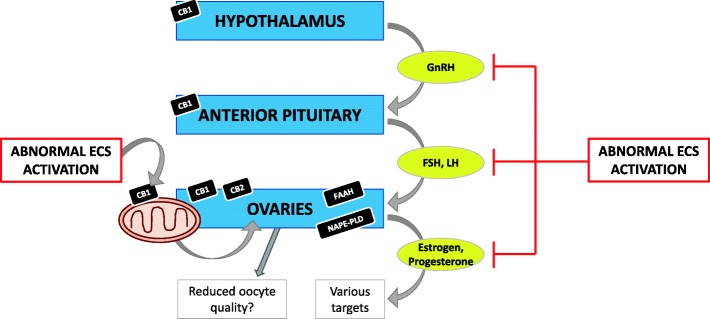
Summary of the major effects of the ECS on the HPO axis. High levels of endocannabinoids and exogenous cannabinoids suppress the release of gonadotropin releasing hormone (GnRH), luteinizing hormone (LH), follicle stimulating hormone (FSH), estrogen and progesterone. Mitochondrial function is associated with oocyte quality. The presence of CB1 on the outer mitochondrial membrane may perturb ovarian function and subsequently oogenesis. Black boxes indicate components of the ECS that have been identified within the anatomical structures represented by each of the blue boxes. Yellow ovals indicate hormones released along the HPO axis when unperturbed. When each of the anatomical structures are perturbed by cannabinoids, the release of the hormones in the yellow ovals is prevented

The presence of the ECS has been demonstrated in numerous cell types where both endogenous and exogenous cannabinoids are associated with the regulation of female reproductive events [9]. Furthermore, the ECS has been localized to areas of the hypothalamus responsible for producing hormones such as GnRH [34], which act through the hypothalamic-pituitary-ovarian (HPO) axis to control a number of aspects of the female reproductive processes. Overall, the effectors of the ECS exert a strong impact on fertility, reproduction and endocrine function [37], as demonstrated by rodent, primate, and human studies [1]. This may account for the effects of cannabis and THC on several aspects of reproductive physiology, including the release of hormones along the HPO axis (Fig. 1), readiness for fertilization and implantation [17, 37, 38]. Chronic exposure to cannabinoids in male rodents and humans has been shown to result in reduced sperm count [34], serum testosterone levels [39] and serum luteinizing hormone (LH) [34]. In females, chronic exposure to cannabinoids has been shown to delay sexual maturation, cause menstrual cycle disruption, depress ovarian follicular maturation, and reduce serum concentrations of LH and sex hormones [34]. Endocannabinoids, their metabolic enzymes, and target receptors have been shown to respond to endocrine signals [9]. Likewise, they have been shown to interfere with reproductive signals in both male and female reproductive processes [9]. It is worth noting that in vivo levels of endocannabinoids, including that within reproductive organs, are tightly regulated by the enzymes that control their metabolism, and when perturbed by exogenous cannabinoids [37], may lead to reproductive failure [36, 37].

## ECS and the HYPOTHALMIC-pituitary-ovarian axis

The events of the ovarian cycle are controlled by an interplay of hormones secreted by three structures, the hypothalamus, anterior pituitary, and ovary, together known as the HPO (or gonadal, HPG) axis [40]. The HPO axis controls the processes involved in oogenesis [38]. The hypophysiotropic hormone sequence along the HPO axis follows a three-hormone sequence: (1) a hypophysiotropic hormone controls the secretion of (2) an anterior pituitary hormone, which then controls the secretion of (3) a hormone from an endocrine gland. This last hormone is permitted to act on its target cells [34, 38]. Within this context, GnRH is released in a pulsatile manner from hypothalamic neuroendocrine cells, which then stimulate specific nuclei in the anterior pituitary gland to produce and secrete two peptide hormones – follicle stimulating hormone (FSH) and LH. Collectively termed gonadotropic hormones (or gonadotropins), FSH and LH regulate the growth and development of the follicle and oocyte and stimulate the ovaries to secrete the sex hormones estrogen and progesterone [40–42] (Fig. 1).

The basic unit of the ovary is the follicle, whose function is to provide support to the oocyte as it passes through a series of distinct stages of development [41, 42]. Depending on the stage of development, follicles differ regarding their responsiveness to gonadotropic hormones. During follicular development, they progress from primordial, primary, then ultimately secondary follicles, during which the follicles are not responsive to gonadotropic hormones because they do not possess functional gonadotropin receptors [41, 42]. Following this, the follicles transition to tertiary follicles (or antral follicles; defining a fluid-filled cavity, similar in composition to that of blood serum [41], adjacent to the oocyte), at which point they become sensitive to/dependent on gonadotropic hormones [41, 42]. Hereafter, FSH significantly increases the rate of growth, while a surge in LH allows the mature oocyte (in the pre-ovulatory follicle) to rupture, releasing the oocyte for fertilization [41, 42]. Estrogen is then synthesized in significant amounts from the pre-ovulatory follicle resulting in a midcycle LH surge. Large amounts of progesterone are subsequently released from the remaining follicular cells which form the corpus luteum, allowing the endometrium to be receptive to a fertilized oocyte [41–43]. For more extensive reviews, see references [41–43].

The ECS has been closely associated with the HPO axis; CB1 receptors have been identified in the hypothalamus and anterior pituitary, and CB1/CB2 receptors are present in the ovary [1, 17, 34]. Other components of the ECS, such as AEA and FAAH have also been expressed in other tissues of the female reproductive system, including the ovaries, oviducts, endometrium and myometrium [1]. The observation that cannabis derivatives induced changes in reproductive processes led researchers to further study the impact of endocannabinoids on the HPG axis, an effect that is seen in both sexes and across various species. These changes include reduced circulating GnRH, anovulatory cycles, and prolonged follicular phase thus delaying ovulation [38]. Most reports suggest that these effects are due to hypothalamic dysfunction; however, the precise mechanisms are not clear [34, 38], whilst others suggest that the effects may be mediated at the pituitary or ovarian levels [34]. Furthermore, the presence of CB1 on the outer mitochondrial membrane [14] and the association between mitochondrial function and oocyte quality [29], suggests that the disruption of endocannabinoid-dependent regulation of oogenesis by THC or other cannabinoids may result in adverse fertility outcome (Fig. 1). Direct mechanistic support for this hypothesis is currently lacking.

A main function of CB1 receptors in the CNS is to regulate the release of various neurotransmitters, such as glutamate and gamma-aminobutyric acid (GABA) [44]. It has been shown that cannabinoids indirectly modify GnRH secretion by reducing the activity of neurotransmitters which facilitate GnRH secretion (i.e. glutamate) whilst stimulating the activity of those known to down regulate GnRH secretion (i.e. GABA) [45, 46]. Using immortalized hypothalamic GnRH neurons (GT1 cells), Gammon et al demonstrated the presence of a complete and functional ECS, and by carrying out perfusion experiments with a CB1 agonist, WIN 55,212–2, completely blocked pulsatile secretion of GnRH [34]. Thus, stimulation of hypothalamic CB1 results in a reduction in the release of GnRH, preventing anterior pituitary stimulation [34]. Scorticati et al also demonstrated, using male and ovariectomized female rats, that administration of AEA intracerebroventricularly resulted in the inhibition of the hypothalamic release of GnRH [47]. Furthermore, Bálint et al., using gonadally intact transgenic female mice tagged with GnRH-GFP (green fluorescent protein) in electrophysiological experiments, also demonstrated that in response to 2-AG/CB1 agonism, the electrical activity of GnRH neurons was repressed [48]. Consistent with this, Chakravarty et al reported that THC treatment lowered GnRH concentration in the hypothalamus of female rats [49]. Taken together, these data show that cannabinoids exert negative effects on reproduction by reducing the secretion of GnRH, which subsequently blocks the release of FSH and LH from the anterior pituitary gland, thus suppressing gonadal function which negatively impacts gonadal estrogen and progesterone release (Fig. 1).

## Role of the ECS in ovarian function

The ovary has two main purposes: to produce female gametes and to secrete various endocrine factors such as estrogen and progesterone [17, 42]. Estrogen enhances the responsiveness of the follicles to gonadotropic hormones and signals the release of GnRH [42, 48], while progesterone, mostly expressed by the corpus luteum [42], slows ovarian follicular growth [50]. In response to the gonadotropins, FSH and LH, these hormones work together to ensure successful oocyte development [17].

While components of the ECS have been identified in female reproductive fluids and in plasma [51], the majority of our knowledge about the effects of cannabinoids on ovarian function is derived from in vitro and animal studies, as well as studies in cannabis users. In 2009, El-Talatini et al reported the presence of the entire ECS within the ovary [1]. Using immunohistochemical staining, CB1, CB2, FAAH, and NAPE-PLD were shown to be localized within human ovarian follicles. They also presented data which suggests that AEA acts by autocrine mechanisms in the follicular cells to stimulate changes that have yet to be determined [1]. Interestingly, CB2 was present at greater levels in the ovarian follicles as compared to CB1 [1]. This finding may suggest a greater immunological role for the ECS in ovarian function. Moreover, FAAH and NAPE-PLD were found to be expressed in the secondary and tertiary follicles, the corpus luteum and corpus albicans, which suggests that AEA may be produced by developing follicles, but not from oocytes, thus serving a role in folliculogenesis [1].

Though AEA fluctuations occur during the ovarian cycle, a surge is reported in the ovary leading up to the time of ovulation, making it possible that endocannabinoid signaling may help to regulate follicular maturation and development [1]. As an example, high intrafollicular levels of AEA allow for ovulation, while the plasma and intrauterine levels must be lowered to allow for implantation of a fertilized oocyte [9]. However, excessively high levels of cannabinoids can impair the processes leading up to and including ovulation, acting not only at the hypothalamic level, but also at the level of the ovarian granulosa cells. Perturbations to the biosynthesis and/or degradation of AEA leading to a net increase in the concentration of AEA has been associated with several human pathologies, including neuroinflammatory diseases, eating disorders [7, 52], and polycystic ovarian syndrome [52, 53].

**Fig. 2:**
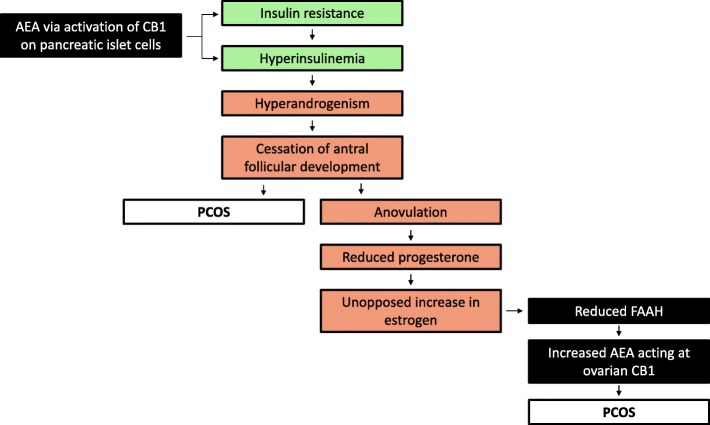
Proposed involvement of the ECS in the pathogenesis of PCOS. The main pathogenic factors relating to PCOS and the influence of some ECS components are illustrated. Black boxes indicate components of the ECS; green boxes indicate metabolic components; orange boxes indicate endocrine/reproductive components

AEA has been identified in ovarian follicular fluids obtained at the time of oocyte retrieval for in vitro fertilization (IVF), which further suggests that it may play a role in follicular/oocyte maturation [9]. In vitro studies using rat granulosa cells have demonstrated that THC exerts a direct inhibitory effect on folliculogenesis and ovulation [1]. Ovulation is dependent on the accumulation of cAMP, an effect that is inhibited by THC, as demonstrated in rat follicular cells [54]. In support of these findings, in humans, a direct negative effect on the ovary has been demonstrated since occasional [17] and heavy-moderate [38] cannabis users present with anovulatory cycles leading to primary infertility. However, the development of tolerance in chronic cannabis users needs to be considered, as this can present as a source of variability in interpreting the data [55]. Furthermore, when these cannabis users undergo IVF treatment, they produce poor quality oocytes and have lower pregnancy rates when compared to non-cannabis users [1]. Interestingly, El-Talatini et al have identified an optimal cut-off point of AEA concentrations in follicular fluid, at 1.09 nM [1], which positively correlated with follicular size and the presence of a mature oocyte. AEA concentrations were higher in follicles in which mature oocytes were retrieved, suggesting that AEA is involved in the maturation of the follicles and/or the oocyte [1]. Mechanistically, estrogen, produced by developing follicles has been shown to inhibit FAAH resulting in increased AEA signaling [36], contributing to its role in folliculogenesis and ovulation. Following ovulation, estrogen levels decline [43], thus releasing the inhibition on FAAH, so it follows that AEA is enzymatically degraded post-ovulation (Fig. 2). THC, unlike AEA, is slowly metabolized and accumulates within adipose tissue, thus mimicking a situation of excess endocannabinoids, or when re-uptake or degradation of the endogenous ligands are impaired [36]. Given that AEA is an agonist at cannabinoid receptors, one might suppose that THC from cannabis would not serve as a detriment to ovulation. However, THC has been shown in several studies to block the release of LH [6, 56–58], which is critical for ovulation, and this may potentiate the probability of infertility.

Using cultured rat granulosa cells, Adashi et al examined the effects of cannabinoids on ovarian function [59]. Their chief finding was that THC, or its metabolites, inhibited FSH-stimulated accumulation of estrogen and progesterone, along with the FSH-stimulated increase in LH receptors. This direct effect of THC was determined to occur at a point downstream of the formation of cAMP and involves the inhibition of steroidogenesis [59]. Indeed, they found that THC reduced the conversion of pregnenolone to progesterone [59]. These effects were shown to be selective, as THC did not produce a negative impact on cell viability nor protein content [59]. These findings suggest that a perturbed ECS, either by exogenous cannabinoids [59], or elevated serum AEA, both of which act via CB1, may contribute to ovarian dysfunction [52].

## Cannabinoids and ovarian pathologies

### Polycystic ovarian syndrome

Polycystic ovarian syndrome (PCOS) is a common endocrine pathology characterized by oligo-anovulation, hyperandrogenism, and the appearance of polycystic ovaries upon ultrasonography. This condition affects approximately 5–15% of women of reproductive age [17, 60]. Data from epidemiological studies demonstrate that PCOS often affects obese women, most of which present with hyperinsulinemia, which are risk factors associated with type II diabetes [17]. Obesity exaggerates insulin resistance leading to compensatory hyperinsulinemia and increased risk of type II diabetes [61]. These metabolic derangements have important implications in the pathogenesis of PCOS. Increased adiposity leads to an increase in serum leptin, which blocks LH secretion [61], leading to infertility and anovulation [17, 61]. Furthermore, hyperinsulinemia increases ovarian steroid hormone production by blocking LH leading to the cessation of follicular development [61].

Studies on the etiology and pathogenesis of PCOS typically have focussed on the association with metabolic syndrome [60], with few studies to date examining the impact of the ECS on PCOS. Indeed, the cardinal features of PCOS, such as insulin resistance and obesity, might be influenced by the ECS. It is well known that the ECS regulates energy balance by regulating appetite, food intake, and glucose metabolism [52]. Importantly, the presence of AEA has been shown to result in insulin hypersecretion and insulin resistance via activation of CB1 in pancreatic islet cells [53] (Fig. 2). In addition, evidence suggests that the ECS can effect ovarian function via modulation of pathways involved in energy balance and metabolic control [52] since obesity is associated with menstrual irregularities, chronic oligo-anovulation, and infertility [52]. Evidence using animal models of obesity, although limited, suggest a connection between increased adiposity and dysregulation of AEA and 2-AG [62].

A recent study by Cui et al aimed to establish a link between the ECS and PCOS using non-obese subjects [53]. Within the context of the proliferative and secretory phases of the menstrual cycle, the endometrium exhibited significantly reduced levels of FAAH in the subjects with PCOS when compared to non-PCOS infertile women who served as the control subjects. Given that FAAH is the catabolic enzyme for AEA, it is biologically plausible that AEA levels may be elevated in patients who suffer from PCOS [53]. Indeed, in obese and type II diabetic patients, serum levels of AEA and 2-AG are significantly higher than that seen in women of healthy weight [52]. Using real-time PCR, these authors demonstrated that CB1 transcripts from omental adipose tissue were lower in PCOS subjects vs. those without PCOS [52]. However, they did not observe significant differences in adipose tissue CB2 transcript expression between the two groups. Interestingly, Cui et al, report that endometrial CB1 expression levels were not different between the aforementioned groups, nor did it fluctuate during the menstrual cycle [53]. In a subsequent study by Cui et al [63], non-obese women with PCOS were treated with Diane-35 (a mix of antiandrogens and estrogens) and metformin (an oral hypoglycemic) which significantly reversed the increase in plasma AEA levels [63]. These findings support a role of the ECS in non-obese women with PCOS, suggesting that the ECS, mainly via increased serum AEA and reduced endometrial FAAH expression, potentiates the progression of PCOS [63] (Fig. 2). Intriguingly, Cui et al relied on the endometrial expression of FAAH, rather than that which is expressed within the ovary. Endometrial FAAH expression was thought to correlate with serum AEA expression; however, serum AEA does not necessarily correlate to that found within tissues. The proposed reasoning was that FAAH, as the main hydrolase for AEA, fluctuates during the menstrual cycle; however, a larger sample size is required to support their findings, along with a direct analysis of ovarian FAAH expression [63]. Studies in obese women with and without PCOS are warranted to ascertain the impact of the ECS on the pathogenesis of PCOS in this cohort. Taken together, the ECS may be intimately involved with the progression of PCOS, likely due to its key role in energy homeostasis, whose components may be useful clinically as biomarkers of PCOS.

### The ECS: A new target for treating ovarian Cancer?

A significant amount of research focus has been directed towards how cannabinoids, particularly the plant-derived and synthetic cannabinoids, effect the initiation and progression of cancer [64]. Synthetic cannabinoids (such as prescription Marinol®), have been widely used to address the negative symptoms associated with chemotherapy, namely neuropathic pain, loss of appetite, nausea and vomiting [3, 5, 23, 65–67]. Beyond the reported palliative effects of phyto- and synthetic cannabinoids, these molecules are gaining recognition for their role in the pathogenesis of cancer [3]. Indeed, various in vivo and in vitro studies have demonstrated that the endocannabinoid system is able to initiate apoptosis and autophagy, induce cell cycle arrest, mount inflammatory responses against malignant cells, and block angiogenic and metastatic processes [3, 67]. In short, cannabinoids exert a variety of anticancer effects by disturbing the signaling pathways involved in malignant transformation and thus tumour progression [67].

Several mechanisms are thought to mediate the anticancer effects of cannabinoids. It has been suggested that stimulation of TRPV1 or PPARγ, along with inhibition of COX2 might mediate the apoptotic and anti-proliferative effects of AEA and synthetic cannabinoids. Furthermore, several signaling pathways governing cell survival, proliferation, and apoptosis, including p38, MAPK, cAMP, PI3K-PKB, and others are activated by ligand-receptor interactions at the classical cannabinoid receptors [67, 68].

Endocannabinoid effects in different tumour models are highly variable and are likely a result of the differential expression of cannabinoid receptors, along with variability between tissue types. Indeed, reports have suggested that differential expression of cannabinoid receptors between normal and cancerous cells may impact the pathogenesis of a malignant tumour [3]. The current view is that cancerous cells express greater levels of CB1 and/or CB2 as compared to normal cells [3, 69]. This finding has been demonstrated in hepatocellular carcinoma, breast cancer, lymphoma, and prostate cancer cells lines [3]. However, a paucity of data exists regarding the role of the ECS in the pathogenesis of ovarian cancer.

To the best of our knowledge, there is only one study that specifically looked at the ECS in the context of ovarian cancer. This recent study by Messalli et al, utilized histochemical analyses in human ovarian tumours and revealed a tumor grade-dependent expression of CB1 receptors. The benign ovarian tumors displayed weak-moderate expression of CB1, with borderline tumors showing a similar trend. In sharp contrast, invasive tumors showed moderate-strong CB1 expression [69]. Curiously, the authors only analyzed FAAH expression in the borderline phenotype, which paralleled the expression pattern of CB1 [69]. They postulated that while low cannabinoid levels may activate proliferative pathways (in noncancerous cells), a higher concentration results in anti-proliferative and apoptotic events in cancerous phenotypes. Importantly, and as previously stated, the CB1 or CB2 expression in cancer cells, including the increase reported by Messalli et al, does not necessarily correlate with the expression pattern from the healthy tissue of origin [68]. Indeed, it has been reported that cannabinoid receptors and their endogenous ligands are generally upregulated in the cancer phenotype when compared to its non-cancerous phenotype. Such elevated levels of cannabinoid receptor expression permit exogenous cannabinoids, administered at therapeutic doses, to impair tumour progression by inducing apoptosis (reviewed in [68, 70]). Furthermore, the expression of the various components of the ECS is not homogenous across all cancers and the mechanisms are complex, thus by pharmacological or genetic manipulations of the ECS, further investigations into the mechanistic connections between the ECS and cancer progression are warranted [67, 69].

## Conclusions and considerations

An important question that arises from the research summarized in this review is whether components of the ECS can be pharmacologically suppressed or upregulated to achieve favourable outcomes regarding fertility and ovarian pathology. Some evidence to support this line of research and provoke further inquiry include:

1. The ECS is activated with tightly regulated spatial and temporal specificity, details of which are still unfolding [71]. It is particularly important to consider that evaluating the impact of exogenous cannabinoids, using high concentrations, may result in confounding results because of the promiscuity of the ECS components.
2. Plasma and local/tissue levels of various components of the ECS do not necessarily correlate. Further, the system fluctuates alongside the menstrual/ovarian cycle [63]. A better understanding of the ECS/endocrine system would facilitate the establishment of clinical biomarkers for oocyte maturity.
3. An understanding of the multiple interactions among different modulators within the ECS along with the crosstalk that occurs with the HPO axis, will be critical for the development of therapeutic interventions for ovarian pathologies.

The data available to date suggests that the ECS is intimately involved in the central and local control of female reproductive events. Perturbations by exogenous cannabinoids in cannabis may disrupt the homeostatic mechanisms of the ECS in female reproductive processes [37] and lead to infertility through dysregulation of ovarian function. The current evidence delineates the presence of ligands, receptors and metabolic enzymes for the synthesis and degradation of endocannabinoids in the female reproductive tract and presents a complex clinical picture. Given the high prevalence of cannabis use in youth [72], combined with the increasing number of municipalities that are legalizing this plant and its extracts for medical and recreational use, it is critical that we more clearly elucidate its impact on the female reproductive process. Furthermore, there needs to be greater pre-clinical research in ovarian cancer growth within the context of the ECS, with the potential for identifying biomarkers and new approaches for adjuvant therapy to address this neoplasm.

## Abbreviations

2-AG: 2-arachidonoyl glycerol
ABHD-6/− 12: α,β-hydrolase-6 and -12
AEA: N-ararachidonoyl ethanolamine
AIBP: AEA intracellular binding proteins
cAMP: cyclic adenosine monophosphate
CB1/2: cannabinoid receptor 1/2
COX: cyclooxygenase
DAGL: diacylglycerol lipase
ECS: endocannabinoid system
EMT: endocannabinoid membrane transporter
ER: endoplasmic reticulum
FAAH: fatty acid amide hydrolase
FABP: fatty acid binding protein
FSH: follicle stimulating hormone
GABA: gamma-aminobutyric acid
GnRH: gonadotropin releasing hormone
GPCR: G-protein-coupled receptor
HPO/G: hypothalamic-pituitary-ovarian/gonadal
HSP: heat shock protein
IVF: in vitro fertilization
LH: luteinizing hormone
LOX: lipoxygenase
MAG/MAGL: monoacylglycerol / monoacylglycerol lipase
MAPK: mitogen-activated protein kinase
NADA: N-arachidonoyldopamine
NAPE: N-acylphosphatidylethanolamine
NAPE-PLD: N-acylphosphatidylethanolamine-phospholipase D
PCOS: polycystic ovarian syndrome
PI3K-PKB: phosphatidylinositol 3-kinase-protein kinase B
PLC: phospholipase C
PPAR: peroxisome proliferator-activated receptors
THC: Δ9-tetrahydrocannabinol
TRPV1: transient receptor potential vanilloid-1
